# Improving the Prognostic Performance of SUV_max_ in ^18^F-Fluorodeoxyglucose Positron-Emission Tomography/Computed Tomography Using Tumor-to-Liver and Tumor-to-Blood Standard Uptake Ratio for Locally Advanced Cervical Cancer Treated with Concurrent Chemoradiotherapy

**DOI:** 10.3390/jcm9061878

**Published:** 2020-06-16

**Authors:** Gun Oh Chong, Shin Young Jeong, Yoon Hee Lee, Shin-Hyung Park, Hyun Jung Lee, Sang-Woo Lee, Dae Gy Hong, Yoon Soon Lee

**Affiliations:** 1 Department of Obstetrics and Gynecology, School of Medicine, Kyungpook National University, Daegu 41944, Korea; gochong@knu.ac.kr (G.O.C.); yhlee1017@knu.ac.kr (Y.H.L.); obgy1019@hanmail.net (H.J.L.); chssa0220@hanmail.net (D.G.H.); yslee@knu.ac.kr (Y.S.L.); 2 Department of Obstetrics and Gynecology, Kyungpook National University Chilgok Hospital, Daegu 41404, Korea; 3 Department of Nuclear Medicine, School of Medicine, Kyungpook National University, Daegu 41944, Korea; swleenm@knu.ac.kr; 4 Department of Nuclear Medicine, Kyungpook National University Chilgok Hospital, Daegu 41404, Korea; 5 Department of Radiation Oncology, School of Medicine, Kyungpook National University, Daegu 41944, Korea; shinhyungpark@knu.ac.kr; 6 Department of Radiation Oncology, Kyungpook National University Hospital, Daegu 41944, Korea; 7 Department of Obstetrics and Gynecology, Kyungpook National University Hospital, Daegu 41944, Korea

**Keywords:** locally advanced cervical cancer, ^18^F-FDG PET/CT, SUV_max_, tumor-to-liver ratio, tumor-to-blood ratio, prognosis

## Abstract

Objective: We sought to evaluate whether the ^18^F-fluorodeoxyglucose uptake normalization of the primary tumor to both the liver and blood pool and lymph nodes to both the liver and blood pool can enhance the discrimination for prognosis prediction in patients with cervical cancer. Methods: A total of 156 patients with cervical cancer (International Federation of Gynecology and Obstetrics stages IIB–IV) treated with concurrent chemoradiotherapy (CCRT) were enrolled. The maximum standardized uptake value (SUV_max_) of tumor (tSUV_max_) and the lymph node (nSUV_max_) divided by the SUV_mean_ of the liver (tumor-to-liver ratio (TLR) and node-to-liver (NLR)) and blood pool (tumor-to-blood ratio (TBR) and node-to-blood ratio (NBR)) were investigated. Univariate and multivariate analyses of disease-free survival (DFS) and overall survival (OS) were performed using clinical and metabolic parameters. A receiver operating characteristic curve analysis was performed to compare the accuracy of the metabolic parameters. Results: The multivariate analysis revealed that NLR (hazard ratio ((HR): 3.54; 95% confidence interval (CI): 1.53–8.19; *p* = 0.0032) and NBR (HR: 3.38; 95% CI: 1.02–11.19; *p* = 0.0457)) were independent prognostic factors for DFS, while TLR (HR: 4.16; 95% CI: 1.19–14.50; *p* = 0.0252), TBR (HR: 3.01; 95% CI: 1.04–8.70; *p* = 0.0415), NLR (HR: 4.84; 95% CI: 1.58–14.81; *p* = 0.0057), and NBR (HR: 6.87; 95% CI: 1.55–30.54; *p* = 0.0113) were significant prognostic factors for OS. The normalization of tSUV_max_ to the liver or blood pool enhanced the discrimination for prediction of recurrence (tSUV_max_ vs. TLR; *p* = 0.0056 and tSUV_max_ vs. TBR; *p* = 0.0099) and death (tSUV_max_ vs. TLR; *p* < 0.0001 and tSUV_max_ vs. TBR; *p* = 0.0001). Conclusions: The normalization of tSUV_max_ was an independent prognostic factor and improved the discrimination for the prediction of tumor recurrence and death in patients with locally advanced cervical cancer treated with CCRT.

## 1. Introduction

Definitive concurrent chemoradiotherapy (CCRT) using a cisplatin-based regimen is currently the standard treatment for locally advanced cervical cancer (International Federation of Gynecology and Obstetrics (FIGO) stages IIB–IV) [1,2]. The contribution of CCRT to improving survival outcomes in locally advanced cervical cancer has been confirmed in previous studies, with a complete clinical response achieved in 70% to 90% patients [3,4]. However, about one-third of patients with cervical cancer experience recurrence, most such cases developing within two years after the completion of therapy [5]. The accurate prediction of tumor recurrence may help to improve survival outcomes and enable the personalization of treatment.

Various metabolic parameters of ^18^F-fluorodeoxyglucose positron-emission tomography/computed tomography (^18^F-FDG PET/CT) have been reported as prognostic factors, including the maximum standardized uptake value (SUV_max_), metabolic tumor volume (MTV), and total lesion glycolysis (TLG). Several studies have also evaluated the prognostic values of primary tumor SUV_max_ (tSUV_max_), but the results were controversial. Some research suggested that tSUV_max_ can predict the prognosis and treatment response [6,7], whereas other studies cautioned that tSUV_max_ is a poor independent predictor of disease progression, recurrence, and death [8,9].

SUV measurements are the most widely used and generally accepted indices in the published literature for assessing disease activity in various cancers. However, many factors can affect the reliability of SUV, such as the time between injection and imaging acquisition, partial volume effects, extravasation of administered ^18^F-FDG at the site of injection, residual activity in the syringe, decay of the injected dose, and technological characteristics and parameters [10]. However, it has been repeatedly recognized that at least some of these issues can be reduced or eliminated if SUV is normalized to the SUV of a suitable reference region [11]. The liver and blood pool are the most widely used references because they maintain a nearly constant SUV level over time following the injection of ^18^F-FDG [12,13]. Recent studies have shown that the tumor-to–normal liver SUV ratio (TLR) and tumor-to–blood pool SUV ratio (TBR) are independent prognostic factors in several cancers [13,14]. However, the prognostic roles of the normalization of SUV_max_ to the liver or blood pool have not been reported previously in cervical cancer. Therefore, we hypothesized that the normalization of SUV_max_ to the liver or blood pool may enhance the prognostic values of SUV_max_ and predict tumor recurrence and death.

This study sought to evaluate whether the normalization of SUV_max_ to the liver or blood pool can reduce or eliminate the limitations of SUV_max_ and to examine the prognostic role of SUV_max_ in locally advanced cervical cancer treated with CCRT.

## 2. Materials and Methods

### 2.1. Patients

For this study, we enrolled 156 patients with biopsy-confirmed cervical cancer treated with CCRT between September 2005 and November 2017. Retrospective data collection and analysis were approved by the institutional review board of Kyungpook National University Chilgok Hospital, while the need for informed consent was waived because of the retrospective design of the study. Disease staging was conducted according to the FIGO staging system [15]. All patients had previously undergone ^18^F-FDG PET/CT for initial diagnosis, staging, and radiotherapy planning. Patients who exhibited evidence of distant metastatic disease or a history of previous surgery, radiotherapy, or chemotherapy were excluded from the study.

The clinical and pathological parameters were reviewed and retrieved, including age, histology, FIGO stage, tumor size, presence of lymph node metastasis, and serum squamous cell carcinoma (SCC) antigen.

### 2.2. Treatment

All patients were treated with a combination of external beam radiotherapy (EBRT), followed by high-dose-rate (HDR) intracavitary radiotherapy (ICR) with curative intent. EBRT was delivered to the whole pelvis via a three-dimensional conformal radiation therapy four-field box technique (1.8 Gy daily fractions, five times/week, for a total dose of 45 Gy). Extended-field radiotherapy encompassing the pelvis and para-aortic nodal area was administered to patients with para-aortic nodal involvement, and a four-field box technique was similarly used in this case. HDR ICR was initiated after the delivery of an EBRT dose of 39.6 Gy. An additional 5.4 Gy was administered with a midline block. A parametrial boost of 10 Gy in five fractions was also administered to patients with parametrial involvement. ICR was delivered twice per week in five fractions with a fractional dose of 6 Gy at point A. Weekly cisplatin at a dose of 40 mg/m^2^ was administered during radiotherapy. The first course of cisplatin was administered on the first day of radiotherapy.

### 2.3. ^18^F-FDG PET/CT Image Acquisition

All patients fasted for at least six hours, and their blood glucose levels were determined before the administration of ^18^F-FDG. Patients with blood glucose levels higher than 150 mg/dL were rescheduled for a later examination, and treatment was administered to maintain a blood glucose concentration of less than 150 mg/dL in all participants. Patients received intravenous injections of approximately 5.2 MBq of FDG per kilogram of body weight and were advised to rest for one hour before undergoing the acquisition of ^18^F-FDG PET/CT images. The ^18^F-FDG PET/CT scans were performed using Discovery 600 (GE Healthcare, Chicago, IL, USA). Before the PET scan, for attenuation correction, a low-dose CT scan was obtained without contrast enhancement from the skull base to the thigh while the patient was in the supine position and breathing quietly. PET scans were also obtained from the skull base to the thigh at 2.5 min per bed position. PET images were reconstructed with a 128 × 128 matrix and an ordered-subset expectation maximum iterative reconstruction algorithm.

### 2.4. Image Interpretation and PET Data Analyses

The PET/CT images were interpreted by two experienced nuclear medicine physicians, and a final consensus was achieved for all patients. A positive finding was defined as any focus with increased FDG uptake as compared with the surrounding normal tissue. Foci of FDG uptake due to normal physiology or benign variants, such as muscular exercise or an infectious pulmonary infiltration, were excluded from the analysis. The PET images were analyzed visually and semi-quantitatively by measuring the maximum SUV of the primary tumor (pSUV_max_) and metastatic lymph node (nSUV_max_), the TLR, the TBR, the metastatic lymph node–to–normal liver SUV ratio (NLR), and the metastatic lymph node–to–blood pool SUV ratio (NBR). The image display and analysis were achieved using the volume viewer software on an Advantage Workstation 4.5 (GE Medical Systems, Waukesha, WI, USA), which provides a convenient and automatic method by which to delineate the volume of interest (VOI) using an isocontour threshold method based on SUV. For each patient, the nSUV_max_ was designated as the highest SUV_max_ of all metastatic lymph nodes. The mean SUV of the mediastinal background plus two standard deviations was used as the threshold to automatically calculate SUV_max_. If the SUV_max_ of the metastatic lymph node was lower than that of the threshold, we regarded SUV_max_ of the metastatic lymph node as 0. SUV_max_ was obtained using the following formula: SUV_max_ = maximum activity in the region of interest (mBq/g) / (injected dose (mBq)/body weight (g)). The mean SUV of the normal liver was obtained by taking the average of the three VOIs (two in the right lobe and one in the left lobe) of 1 cm in diameter. The mean SUV of the mediastinal blood pool was calculated using a 25-mm-diameter spherical VOI that was manually delineated in the aortic arch of each patient.

### 2.5. Clinical Follow-Up

Clinical follow-up assessments of patients were conducted every three months until two years after treatment, every six months from two years to five years after treatment, and annually thereafter. Failure was defined as a biopsy-proven recurrence or documentation of the progression of disease on serial imaging studies. Failure patterns were divided into four groups: (1) none, (2) isolated local failure that included the central pelvis and/or pelvic lymph nodes, (3) distant failure that included para-aortic and supraclavicular lymph nodes, and (4) combined local and distant failure.

### 2.6. Statistical Analysis

Continuous data are expressed as means ± standard deviations, and categorical data are presented as frequencies and percentages. The time to event was calculated as the time interval from the date of diagnosis to the date at which the first clinical or imaging findings were revealed that suggested disease recurrence. The differences between subsets were evaluated using a Student’s *t*-test, while differences between proportions were compared using the chi-squared test.

To identify the optimal cutoff of continuous variables for the prediction of recurrence and death, a receiver operating characteristic (ROC) curve analysis was performed. Meanwhile, the Kaplan–Meier method and the log-rank test were adopted in the survival analysis of prognostic factors, while a Cox proportional hazards model was used to evaluate prognostic variables for univariate and multivariate comparisons, and the estimated hazard ratios (HRs) are presented with 95% confidence intervals (CIs). The MedCalc version 19.2 statistical software program (MedCalc Software Ltd., Ostend, Belgium; https://www.medcalc.org; 2020) was used for all statistical analyses. A *p*-value of less than 0.05 was considered to be statistically significant.

## 3. Results

### 3.1. Clinical Features and Treatment Outcomes

The clinical characteristics of the study participants are listed in Table 1. The mean age of the patients was 55.1 ± 12.9 years. The predominant FIGO stage was IIB (76.9%), followed by IIIB (10.3%), IIIA (9.0%), and IVA (3.8%). A total of 102 patients (65.4%) had metastases to the regional lymph node. The mean tSUV_max_, TLR, and TBR were 6.0 ± 2.5, 5.9 ± 4.4, and 9.5 ± 6.0, and the mean nSUV_max_, NLR, and NBR were 3.3 ± 7.3, 2.3 ± 4.0, and 3.1 ± 5.0, respectively. After a median follow-up of 49.5 months (range: 4–157 months), 55 patients (35.3%) had experienced recurrence and 30 patients (19.2%) had died of disease progression. Of the 55 patients who experienced disease recurrence, 17 showed local recurrence only, 29 showed distant recurrence only, and nine showed both local and distant recurrence.

### 3.2. Cutoff Value of Metabolic Parameters

The optimal cutoff values of tSUV_max_, TLR, and TBR for predicting tumor recurrence were 4.81, 4.99, and 6.96, respectively (area under the curve (AUC) = 0.506, *p* = 0.6433 for tSUV_max_; AUC = 0.610, *p* = 0.0041 for TLR; and AUC = 0.607, *p* = 0.0071 for TBR). The optimal cutoff values calculated for nSUV_max_, NLR, and NBR for predicting tumor recurrence were 4.63, 2.02, and 1.97, respectively (AUC = 0.732, *p* < 0.0001 for nSUV_max_; AUC = 0.726, *p* < 0.0001 for NLR; and AUC = 0.722, *p* < 0.0001 for NBR) (Supplementary Materials: Table S1).

The optimal cutoff values for tSUV_max_, TLR, and TBR for predicting death were 6.43, 5.14, and 7.52, respectively (AUC = 0.523, *p* = 0.2636 for tSUV_max_; AUC = 0.688, *p* < 0.0001 for TLR; and AUC = 0.686, *p* < 0.0001 for TBR). The optimal cutoff values calculated for nSUV_max_, NLR, and NBR for predicting death were 4.91, 2.61, and 3.07, respectively (AUC = 0.724, *p* < 0.0001 for nSUV_max_; AUC = 0.735, *p* < 0.0001 for NLR; and AUC = 0.740, *p* < 0.0001 for NBR) (Table S1).

### 3.3. Univariate and Multivariate Survival Analyses

In the univariate analysis, the FIGO stage (HR: 4.64; 95% CI: 2.31–9.32; *p* < 0.0001), tumor size (HR: 2.48; 95% CI: 1.41–4.34; *p* = 0.0016), lymph node metastasis (HR: 3.09; 95% CI: 1.79–5.32; *p* < 0.0001), serum SCC (HR: 3.71; 95% CI: 1.94–7.12; *p* = 0.0001), TLR (HR: 2.26; 95% CI: 1.37–3.87; *p* = 0.0031), TBR (HR: 2.23; 95% CI: 1.31–3.81; *p* = 0.0033), nSUV_max_ (HR: 6.86; 95% CI: 3.81–12.36; *p* < 0.0001), NLR (HR: 6.26; 95% CI: 3.51–11.14; *p* < 0.0001), and NBR (HR: 4.74; 95% CI: 2.76–8.15; *p* < 0.0001) were significant prognostic factors for disease-free survival (DFS). For the prediction of death, the age (HR: 2.24; 95% CI: 1.06–4.77; *p* = 0.0355), FIGO stage (HR: 7.30; 95% CI: 2.85–18.69; *p* < 0.0001), tumor size (HR: 3.09; 95% CI: 1.50–6.38; *p* = 0.0023), lymph node metastasis (HR: 2.96; 95% CI: 1.41–6.18; *p* = 0.0039), paraaortic lymph node metastasis (HR: 2.78; 95% CI: 1.10–6.99; *p* =0.0305), serum SCC (HR: 3.75; 95% CI: 1.55–9.08; *p* = 0.0035), TLR (HR: 4.23; 95% CI: 2.05–8.70; *p* = 0.0001), TBR (HR: 4.26; 95% CI: 2.07–8.79; *p* = 0.0001), nSUV_max_ (HR: 7.72; 95% CI: 3.53–16.88; *p* < 0.0001), NLR (HR: 10.27; 95% CI: 4.51–23.39; *p* < 0.0001), and NBR (HR: 8.02; 95% CI: 3.71–17.36; *p* < 0.0001) were significant prognostic factors (Table 2).

**Figure 1:**
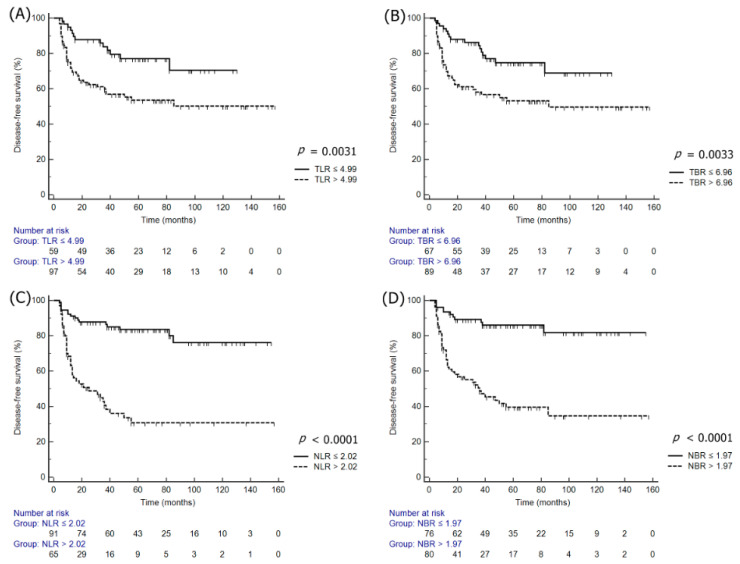
Kaplan–Meier survival plots of DFS according to the normalized tSUV_max_ and nSUV_max_ to the liver or blood: (**A**) TLR, (**B**) TBR, (**C**) NLR, and (**D**) NBR.

**Figure 2:**
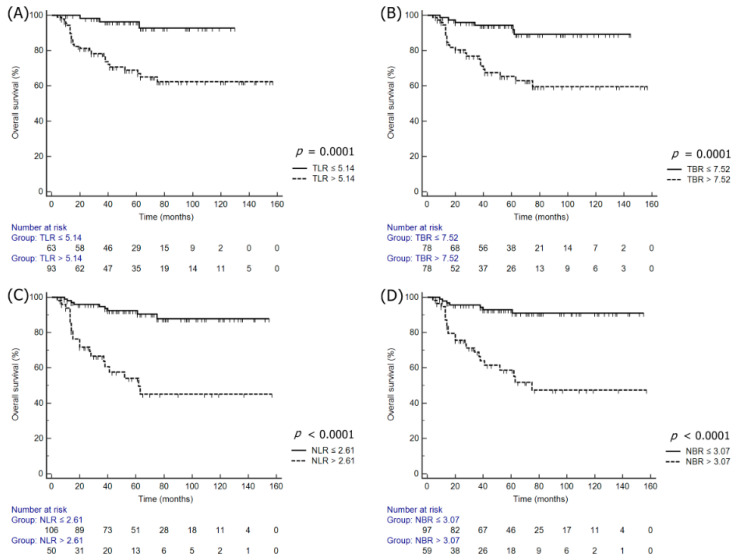
Kaplan–Meier survival plots of OS according to the normalized tSUV_max_ and nSUV_max_ to the liver or blood: (**A**) TLR, (**B**) TBR, (**C**) NLR, and (**D**) NBR.

The multivariate analysis performed for DFS and overall survival (OS) following the adjustment for the effects of clinicopathologic variables, which were statistically significant in the univariate survival analyses, revealed that nSUV_max_ (HR: 4.06; 95% CI: 1.74–9.44; *p* = 0.0012), NLR (HR: 3.54; 95% CI: 1.53–8.19; *p* = 0.0032), and NBR (HR: 3.38; 95% CI: 1.02–11.19; *p* = 0.0457) were independent prognostic factors for DFS, while TLR (HR: 4.16; 95% CI: 1.19–14.50; *p* = 0.0252), TBR (HR: 3.01; 95% CI: 1.04–8.70; *p* = 0.0415), nSUV_max_ (HR: 6.99; 95% CI: 1.56–31.25; *p* = 0.0109), NLR (HR: 4.84; 95% CI: 1.58–14.81; *p* = 0.0057), and NBR (HR: 6.87; 95% CI: 1.55–30.54; *p* = 0.0113) were independent prognostic factors for OS (Table 3). The Kaplan–Meier survival plots revealed significant differences in DFS and OS when stratified by TLR, TBR, NLR, and NBR (Figure 1 and Figure 2).

### 3.4. Comparison ROC for the Prediction of Tumor Recurrence and Death

**Figure 3:**
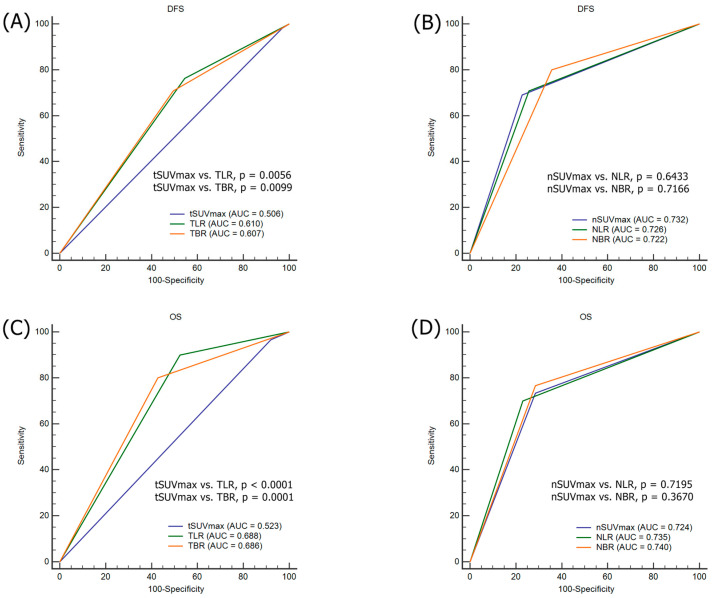
Comparison of ROC curve analyses. (**A**) tSUV_max_ versus TLR and TBR for DFS. (**B**) nSUV_max_ versus NLR and NBR for DFS. (**C**) tSUV_max_ versus TLR and TBR for OS. (**D**) nSUV_max_ versus NLR and NBR for OS.

To enhance the discrimination for the prediction of tumor recurrence and death, SUV_max_ was normalized to the liver and blood pool. TLR and TBR showed significant improvements in discrimination for tumor recurrence when compared with tSUV_max_ (tSUV_max_ vs. TLR; *p* = 0.0056, and tSUV_max_ vs. TBR; *p* = 0.0099) (Figure 3A). Furthermore, TLR and TBR demonstrated significant improvements in the accuracy of risk prediction for OS relative to tSUV_max_ (tSUV_max_ vs. TLR; *p* < 0.0001, and tSUV_max_ vs. TBR; *p* = 0.0001) (Figure 3C). However, the normalization of the nSUV_max_ did not improve the discrimination for tumor recurrence and death (Figure 3B,D).

## 4. Discussion

There are two major findings in this study. First, the normalization of tSUV_max_ to the liver or blood pool enhanced the level of discrimination for the prediction of tumor recurrence and death in locally advanced cervical cancer treated with CCRT. Second, TLR, TBR, NLR, and NBR are independent prognostic factors for predicting survival outcomes.

Several studies have demonstrated that pretreatment tSUV_max_ can predict survival outcomes. The largest retrospective study of 287 patients with FIGO stages IA2 to IVB cervical cancer established the following three prognostic groups based on tSUV_max_ with five-year OS rates: 95% for a tSUV_max_ of 5.2 or less, 70% for a tSUV_max_ between 5.2 and 13.3, and 44% for a tSUV_max_ greater than 13.3 [6]. Moreover, in a recent multicenter retrospective study that enrolled 270 patients with locally advanced cervical cancer treated with CCRT, a tSUV_max_ of 12 or greater was an independent prognostic biomarker for predicting tumor recurrence (HR: 2.14; 95% CI: 1.32–3.47; *p* = 0.002) and death (HR: 3.06; 95% CI: 1.46–6.44; *p* = 0.003) [16].

However, other studies have shown that tSUV_max_ is not an independent prognostic metabolic parameter. A study of 53 patients with FIGO stages IB1 to IVA determined the optimal tSUV_max_ cutoff to be 16.0; however, this did not significantly predict the progression or DFS [8]. SUV_max_ has several limiting factors, such as it representing the highest metabolic voxel but not the metabolic activity of the entire tumor. Meanwhile, research suggests volume-based metabolic parameters such as MTV and TLG are of prognostic value in cervical cancer [17]. However, MTV and TLG might include inflammation around the primary tumor associated with weakness [18]. SUV_max_ exhibits a high degree of reproducibility and is a less time-consuming method to apply. Despite these advantages, SUV_max_ may not be an adequate surrogate marker for representing the metabolic rate of the tumor, so other metabolic parameters that can predict prognosis should be further explored. The mediastinum vessel and normal liver tissue are the most frequent candidates among normal tissues [19].

Among various metabolic parameters, the tSUV_max_ normalized to liver uptake after neoadjuvant chemotherapy was the best predictor of a pathologic complete response in locally advanced rectal cancer [13]. Moreover, the standardized uptake TBR was an independent predictor of recurrence in non-small-cell lung cancer [14]. In our study, tSUV_max_ had no significant impact in predicting tumor recurrence or death. After the normalization of tSUV_max_ to the liver or blood pool, however, tSUV_max_ demonstrated a significant prognostic performance. We confirmed the improvement in discrimination for the prediction of tumor recurrence and death using the normalization of tSUV_max_ to the liver or blood pool by comparing ROC curves among tSUV_max_, TLR, and TBR. Moreover, the normalization of tSUV_max_ to the liver or blood pool showed a significant prognostic value for predicting tumor recurrence or death in univariate and multivariate analyses. These enhancements of the prognostic value may make it possible to personalize treatment in a way that may involve higher doses of radiation boosts, consolidation chemotherapy, or adjuvant hysterectomy.

The lymph node status on ^18^F-FDG PET/CT images has also been shown to predict tumor recurrence and survival outcomes in patients with cervical cancer. A prospective cohort study of 560 patients who underwent pretreatment ^18^F-FDG PET/CT imaging demonstrated that patients with PET-positive lymph nodes had significantly worse DFS as compared with those with PET-negative nodes [20]. Furthermore, it has also been documented that the level of nSUV_max_ correlates with survival outcomes [7,21]. Only a limited number of studies to date have evaluated the prognostic roles of NLR or NBR. Furthermore, only one study has evaluated the normalization of nSUV_max_ to the liver. Higher NLR on pretreatment ^18^F-FDG PET/CT images was shown to be an independent prognostic factor for worse distant metastasis-free survival in locally advanced nasopharyngeal carcinoma [22]. Similarly to our previous research [9,16], we found in the current study that nSUV_max_ was a powerful biomarker for predicting tumor recurrence and death and that NLR and LBR were significant prognostic factors. However, normalized nSUV_max_ to the liver or blood pool did not demonstrate superiority over conventional nSUV_max_ for predicting tumor recurrence or survival.

Our study has some noteworthy limitations that must be considered. First, it was a retrospective study including a limited number of patients. Second, a histopathological verification of the lymph nodes was not performed. Finally, some variables tended to have a wider 95% CI than *p*-values, especially when considering the OS. The wider 95% CI width resulted in a relatively small sample and event size in some variables. A larger patient cohort study involving multi-institutional cooperative groups is needed to improve the statistical results.

Despite these limitations, our study offers some unique and significant findings and differs from previous studies in several aspects. To the best of our knowledge, this is the first study to evaluate how the normalization of tSUV_max_ and nSUV_max_ to the liver or blood pool affects prognostic values in cervical cancer. Metabolic parameters that did not demonstrate a prognostic role had a significant prognostic value for predicting tumor recurrence and death when normalized to the liver or blood pool. Moreover, this is the first research to demonstrate the prognostic value of TLR, TBR, NLR, and NBR in locally advanced cervical cancer treated with CCRT. TLR, TBR, NLR, and NBR were found to be independent prognostic factors for both DFS and OS.

## 5. Conclusions

Normalized tSUV_max_ and nSUV_max_ to the liver or blood pool showed a significant prognostic value in patients with locally advanced cervical cancer treated with CCRT. Moreover, the normalization of metabolic parameters improved the level of discrimination for the prediction of tumor recurrence and death, especially in the case of tSUV_max_. The normalization of metabolic parameters to the liver or blood pool may enable the standardization of metabolic parameters for multicenter studies.

## Figures and Tables

**Table 1:** Clinicopathologic characteristics and PET metabolic parameters of patients with and without recurrence.

Variables	All (*n* = 156)	No Recurrence (*n* = 101)	Recurrence (*n* = 55)	*p*-Value
Age (years)	55.1 ± 12.9	55.5 ± 12.8	54.3 ± 13.0	0.5965
Histology (*n*, %)				0.1244
SCC	141 (90.4)	94 (93.1)	47 (85.5)	
AC/ASC	15 (9.6)	7 (6.9)	8 (14.5)	
FIGO stage (*n*, %)				0.0006
IIB	120 (76.9)	87 (86.1)	33 (60.0	
IIIA	14 (9.0)	8 (7.9)	6 (10.9)	
IIIB	16 (10.3)	5 (5.0)	11 (20.0)	
IVA	6 (3.8)	1 (1.0)	5 (9.1)	
Tumor size (cm)	4.6 ± 1.5	4.4 ± 1.4	5.1 ± 1.6	0.0080
LN metastasis (*n*, %)	102 (65.4)	55 (54.5)	47 (85.5)	0.0001
Paraaortic LN metastasis (*n*, %)	25 (16.0)	18 (17.8)	7 (12.7)	0.4088
SCC antigen (ng/mL)	20.8 ± 36.0	16.8 ± 35.6	28.1 ± 35.9	0.0715
tSUV_max_	6.0 ± 2.5	6.0 ± 2.7	6.0 ± 2.1	0.9542
TLR	5.9 ± 4.4	6.8 ± 4.1	7.5 ± 4.8	0.3356
TBR	9.5 ± 6.0	9.2 ± 6.0	10.1 ± 6.1	0.3717
nSUV_max_	3.3 ± 7.3	3.5 ± 5.1	7.9 ± 9.5	0.0003
NLR	2.3 ± 4.0	1.5 ± 2.5	3.8 ± 5.6	0.0008
NBR	3.1 ± 5.0	2.1 ± 3.4	5.0 ± 6.7	0.0005

AC = adenocarcinoma; ASC = adenosquamous cell carcinoma; FIGO = International Federation of Gynecology and Obstetrics; LN = lymph node; NBR = node-to-blood ratio; NLR = node-to-liver ratio; nSUV_max_ = nodal maximum standardized uptake value; SCC = squamous cell carcinoma; SUV_max_ = maximum standardized uptake value; TBR = tumor-to-blood ratio; TLR = tumor-to-liver ratio; tSUV_max_ = tumor maximum standardized uptake value.

**Table 2:** Univariate analyses of clinical variable and quantitative metabolic parameters for DFS and OS.

Variables		Disease-Free Survival				Overall Survival		
	Level	HR	95% CI	*p*-Value	Level	HR	95% CI	*p*-Value
Age (years)	>49	1.00	-	-	>49	1.00	-	-
	≤49	1.25	0.70–2.23	0.4584	≤49	2.24	1.06–4.77	0.0355
Histology	SCC	1.00	-	-	SCC	1.00	-	-
	AC/ASC	2.04	0.78–4.98	0.1531	AC/ASC	1.57	0.46–5.35	0.4699
FIGO stage	IIB	1.00	-	-	IIB	1.00	-	-
	>IIB	4.64	2.31–9.32	<0.0001	>IIB	7.30	2.85–18.69	<0.0001
Tumor size	<4 cm	1.00	-	-	<4.2 cm	1.00	-	-
	≥4 cm	2.48	1.41–4.34	0.0016	≥4.2 cm	3.09	1.50–6.38	0.0023
LN metastases	Negative	1.00	-	-	Negative	1.00	-	-
	Positive	3.09	1.79–5.32	<0.0001	Positive	2.96	1.41–6.18	0.0039
Paraaortic LN metastases	Negative	1.00	0.71–2.85	0.3245	Negative	1.00	1.10–6.99	0.0305
	Positive	1.42			Positive	2.78		
Serum SCC	<19.3	1.00	-	-	<19.3	1.00	-	-
	≥19.3	3.71	1.94–7.12	0.0001	≥19.3	3.75	1.55–9.08	0.0035
tSUV_max_	≤4.81	1.00	-	-	≤6.43	1.00	-	-
	>4.81	1.39	0.26–7.30	0.6975	>6.43	2.05	0.60–7.03	0.2528
TLR	≤4.99	1.00	-	-	≤5.14	1.00	-	-
	>4.99	2.26	1.37–3.87	0.0031	>5.14	4.23	2.05–8.70	0.0001
TBR	≤6.96	1.00	-	-	≤7.52	1.00	-	
	>6.96	2.23	1.31–3.81	0.0033	>7.52	4.26	2.07–8.79	0.0001
nSUV_max_	≤4.63	1.00	-	-	≤4.91	1.00	-	-
	>4.63	6.86	3.81–12.36	< 0.0001	>4.91	7.72	3.53–16.88	< 0.0001
NLR	≤2.02	1.00	-	-	≤2.61	-	-	
	>2.02	6.26	3.51–11.14	< 0.0001	>2.61	10.27	4.51–23.39	<0.0001
NBR	≤1.97	1.00	-	-	≤3.07	1.00	-	-
	>1.97	4.74	2.76–8.15	< 0.0001	>3.07	8.02	3.71–17.36	<0.0001

AC = adenocarcinoma; ASC = adenosquamous cell carcinoma; CI = confidence interval; FIGO = International.

**Table 3:** Multivariate analyses of PET parameters in relation to DFS and OS after adjusting for clinicopathologic factors.

Variables	Disease-Free Survival				Overall Survival			
	Level	HR	95% CI	*p*-Value	Level	HR	95% CI	*p*-Value
tSUV_max_	≤4.81	1.00	-	-	≤6.43	1.00	-	-
	>4.81	0.29	0.04–2.38	0.2478	>6.43	0.66	0.08–5.85	0.7117
TLR	≤4.9885	1.00	-	-	≤5.1368	1.00	-	-
	>4.9885	1.48	0.76–2.87	0.2509	>5.1368	4.16	1.19–14.50	0.0252
TBR	≤6.9612	1.00	-	-	≤7.5235	1.00	-	-
	>1.9612	1.42	0.75–2.70	0.2864	>7.5235	3.01	1.04–8.70	0.0415
nSUV_max_	≤4.63	1.00	-	-	≤4.91	1.00	-	-
	>4.63	4.06	1.74–9.44	0.0012	>4.91	6.99	1.56–31.25	0.0109
LNR	≤2.0021	1.00	-	-	≤2.6098	1.00	-	-
	>2.0021	3.54	1.53–8.19	0.0032	>2.6098	4.84	1.58–14.81	0.0057
NBR	≤1.9681	1.00	-	-	≤3.0705	1.00	-	-
	>1.9681	3.38	1.02–11.19	0.0457	>3.0705	6.87	1.55–30.54	0.0113

CI = confidence interval; FIGO = International Federation of Gynecology and Obstetrics; HR = hazard ratio; NBR = node-to-blood ratio; NLR = node-to-liver ratio; nSUV_max_ = nodal maximum standardized uptake value; SUV_max_ = maximum standardized uptake value; TBR = tumor-to-blood ratio; TLR = tumor-to-liver ratio; tSUV_max_ = tumor maximum standardized uptake value.

## Supplementary Materials

The following are available online at https://www.mdpi.com/2077-0383/9/6/1878/s1, Table S1. Comparison of receiver operating characteristic curve analyses.

## Abbreviations

CCRT	concurrent chemoradiotherapy
CI	confidence interval
DFS	disease-free survival
^18^F-FDG PET/CT	F-18 fluorodeoxyglucose positron emission tomography/computed tomography
FIGO	Advanced International Federation of Gynecology and Obstetrics
HF	heterogeneity factor
HR	hazard ratio
SUVmax	maximum standardized uptake value
MTV	metabolic tumor volume
ROC	receiver operating characteristic
TLG	total lesion glycolysis
WB	whole body
