# Supplementary material for: Improving the Prognostic Performance of SUVmax in 18F-Fluorodeoxyglucose Positron-Emission Tomography/Computed Tomography Using Tumor-to-Liver and Tumor-to-Blood Standard Uptake Ratio for Locally Advanced Cervical Cancer Treated with Concurrent Chemoradiotherapy
Source: J Clin Med. 2020 Jun 16;9(6):1878. doi: 10.3390/jcm9061878 (PMC7355778; doi:10.3390/jcm9061878)
Supplement: Supplementary file 1 [file jcm-09-01878-s001.pdf]

Supplementary Materials: Improving the Prognostic Performance of SUVmax in ^{18}F-FDG PET/CT Using Tumor-to-Liver and Tumor-to-Blood Standard Uptake Ratio for Locally Advanced Cervical Cancer Treated with Concurrent Chemoradiotherapy

Gun Oh Chong, Shin Young Jeong, Yoon Hee Lee, Shin-Hyung Park, Hyun Jung Lee, Sang-Woo Lee, Dae Gy Hong and Yoon Soon Lee

Table S1. Comparison of receiver operating characteristic curve analyses.

Variables	Cut-off	AUC	95% CI	P value	Sensitivity	Specificity
Disease-free survival						
tSUVmax	>4.81	0.506	0.42–0.59	0.6433	0.982	0.003
TLR	>4.99	0.610	0.53–0.69	0.0041	0.764	0.455
TBR	>6.96	0.607	0.53–0.68	0.0071	0.709	0.505
nSUVmax	>4.63	0.732	0.66–0.80	<0.0001	0.691	0.772
NLR	>2.02	0.726	0.65–0.79	<0.0001	0.709	0.743
NBR	>1.97	0.722	0.64–0.79	<0.0001	0.800	0.644
Overall survival						
tSUVmax	>6.43	0.523	0.44–0.60	0.2636	0.967	0.008
TLR	>5.14	0.688	0.61–0.76	<0.0001	0.900	0.476
TBR	>7.52	0.686	0.61–0.76	<0.0001	0.800	0.571
nSUVmax	>4.91	0.724	0.65–0.79	<0.0001	0.733	0.714
NLR	>2.61	0.735	0.66–0.80	<0.0001	0.700	0.770
NBR	>3.07	0.740	0.66–0.81	<0.0001	0.767	0.714

AUC = area under curve; CI = confidence interval; NBT = node-to-blood ratio; NLR = node-to-liver ratio; nSUVmax = nodal maximum standardized uptake value; SUVmax = maximum standardized uptake value; TBR = tumor-to-liver ratio; TLR = tumor-to-liver ratio; tSUVmax = tumor maximum standardized uptake value.
